# What Are the Predictors of Acute Care Nurses' Stroke Knowledge?: Empirical Research Quantitative

**DOI:** 10.1002/nop2.70106

**Published:** 2024-12-06

**Authors:** Catherine T. Leach, Linda P. Bolin, Melvin S. Swanson, Ashley E. Burch, Patricia C. Woltz

**Affiliations:** ^1^ WakeMed Health & Hospitals Adjunct Faculty, East Carolina University, College of Nursing Raleigh North Carolina USA; ^2^ College of Nursing East Carolina University Greenville North Carolina USA; ^3^ Health Services and Information Management East Carolina University Greenville North Carolina USA; ^4^ WakeMed Health & Hospitals Raleigh North Carolina USA

**Keywords:** in‐hospital stroke, nurse, stroke knowledge

## Abstract

**Aims:**

To examine the predictability of categorical and quantitative study variables on acute stroke knowledge amongst study participants.

**Design:**

Non‐experimental, descriptive correlational.

**Methods:**

A convenience sample of registered nurses caring for adult, hospitalized patients recruited from the urban Southeast. A three‐part survey was emailed to the nurses: demographics, stroke education and experience, and the Acute Stroke Management Questionnaire.

**Results:**

Intensive Care Unit and Emergency Department nurses had the highest level of stroke knowledge. Nurses reporting three or more post‐licensure education modalities had higher stroke knowledge scores than those reporting two or less. Practice setting, self‐perception of stroke knowledge, self‐identified knowledge of acute stroke management and three items related to code stroke were significantly correlated with stroke knowledge.

**Conclusion:**

Various methods of training and reinforcement of learning using varying teaching styles are effective in increasing nurses' stroke knowledge. Further stroke education is needed for nurses across the acute care setting.

**Implications:**

Future studies should explore lived experiences of nurses to gain understanding of nonquantifiable factors that nurses feel have strengthened their stroke knowledge.

**Impact:**

- What Problem Did the Study Address?
  ○ Addressing the gap in the knowledge of predictors of nurse stroke knowledge.

**Main Findings?:**

Practice setting, post‐licensure stroke education, code stroke familiarity, confidence and understanding were all predictive of the overall stroke knowledge.

**Whom Will the Research Have an Impact?:**

Nurses, nurse educators, patients experiencing and at risk for stroke.

**Reporting Method:**

The STROBE checklist was used to strengthen the reporting of the results.

**Patient or Public Contribution:**

Nurses voluntarily participated in this study by completing the online questionnaire.

**What Does This Paper Contribute to the Wider Global Clinical Community?:**

- Provides predictors of stroke knowledge for nurse leaders to have a better understanding of nurses' educational needs.
- Provides evidence of a quick, effective measure of nurses' stroke knowledge.

## 1 Introduction

‘Time is brain’ is a phrase coined to describe the importance of a timely, efficient response to stroke symptoms (National Institute of Neurological Disorders and Stroke [NINDS] 2020). This study focuses on ischaemic strokes, as the thrombolytic treatments that result from the ‘time is brain’ and code stroke initiatives are for ischaemic, non‐haemorrhagic strokes. Although recent efforts have been made to improve treatment times for in‐hospital strokes (IHS), these patients are still experiencing high mortality rates and poor outcomes when compared to community occurring strokes (COS) (Chen et al. 2018). Nurses have been shown to play a large role in recognising and treating IHS (Yang et al. 2019; George et al. 2017; Nouh et al. 2022); however, although nurses receive further training upon employment, the everchanging state of the science regarding IHS treatment and resolution presents a challenge for nurses to stay abreast with best practices. Additionally, stroke education and training recommendations for nurses have neither been noted nor standardised.

## 2 Background

There are many non‐modifiable and modifiable risk factors that help prevent strokes, but when they occur, ischaemic strokes are often treatable if the response is timely. Non‐modifiable risk factors include those unable to be intervened on, that is, patient's age, sex, race, ethnicity and past medical history of stroke (Colsch 2021). However, a modifiable risk is the timeliness of recognition of symptom onset and time to treatment and/or intervention (Yang et al. 2019). Each second an ischaemic stroke is left untreated, 32,000 neurons are lost, which accelerates aging by close to 9 h (Saver 2006), and for every missed or untreated stroke, a person will lose over 1 billion neurons and 36 years of life (Saver 2006). It is imperative that nurses intervene with the modifiable risk factor of timeliness of treatment for IHS to preserve the most amount of healthy brain tissue and facilitate positive patient outcomes.

About 20% of strokes will occur while a person is already hospitalised (Chen et al. 2018; Emmett et al. 2019; Lu et al. 2019). The occurrence of IHS can be related to surgical/procedural risks and complications and/or comorbidity risk factors that led to the initial hospitalisation (Yang et al. 2019). Although the presence of an established multidisciplinary team may imply the simplicity of recognition and treatment of IHS when compared to strokes occurring in the community, IHS are often missed or misunderstood for a variety of non‐stroke‐related conditions such as delirium, or reaction to pharmacological agents including narcotics (Chen et al. 2018).

Because of the complexity of identifying and treating IHS, the American Heart Association (AHA) provides a set of guidelines and recommended benchmark timeframes (AHA 2019). The timeframes are used to support the ‘time is brain’ initiative and are set in place to ensure that patients experiencing stroke symptoms have optimal chances of receiving the time‐sensitive interventions to prevent further complications or ischaemic brain tissue (AHA 2019).

To improve benchmark times for stroke treatment and improve patient outcomes, many hospital systems have initiated a ‘code stroke’ system. A code stroke is a systematic response that activates a team of stroke professionals when stroke symptoms are examined in a patient (Yang et al. 2019; Manners et al. 2019). The literature shows that a code stroke system decreases the time from stroke symptom onset to assessment, radiologic imaging and ultimate treatment (Yoo et al. 2016). Certain studies have also noted a nurse‐driven code stroke process as being the most effective way to drive this systematic response (Yang et al. 2019; George et al. 2017). A nurse‐driven code stroke implies that the nurse is at the forefront or the leader of the code stroke team. Nurses have been shown to identify acute strokes more frequently and earlier than physicians or other healthcare team members, which could be attributed to the time nurses spend at the patients' bedsides compared to other team members (Yang et al. 2019; George et al. 2017; Nouh et al. 2022).

Despite effective code stroke initiatives, IHS patients have still shown to have longer time to treatment when compared to COS patients (Chen et al. 2018). This finding is unexpected, as transportation time to the hospital and medical work‐up and triage are eliminated in IHS (Chen et al. 2018). Subsequently, IHS patients experience higher mortality, poorer outcomes and less frequent discharges home when compared to their COS counterparts (Chen et al. 2018; Bekelis et al. 2016; Emmett et al. 2019).

The literature is unclear on recommendations for stroke, IHS and code stroke education for nurses (El Ammar et al. 2020; Yoo et al. 2016). Mellon et al. (2015) explain that the lack of knowledge amongst hospital staff may contribute to the delays in treatment and assessment of IHS. Many public health initiatives have been created and implemented in recent years to promote lay persons' stroke knowledge; however, there are little data explaining stroke knowledge provided to nurses and hospital personnel (Mellon et al. 2015). The AHA recently recommended the education of hospital staff to improve the recognition of stroke and the empowering of staff to act when a stroke is recognised (Nouh et al. 2022). However, the specifics of the education, that is, timeline, frequency, delivery method and required personnel, is omitted. Many studies indicate ongoing and annual stroke education to nurses and staff working on neuro‐specific or stroke floors; while important, IHS have been shown to occur most frequently on cardiac/cardiovascular and post‐operative floors, not stroke floors (Manners et al. 2019; Lu et al. 2019; Chen et al. 2018).

Nurses receive initial stroke education through their pre‐licensure programmes; however, stroke guidelines and best practices constantly evolve. This, in addition to the time‐sensitive component, results in unrealistic expectations for nurses to stay current with the guidelines without proper continuing education. The benchmark timeframes outlined by the AHA and the effectiveness of code stroke processes can only be achieved if nurses are knowledgeable and aware of IHS and the protocols in place.

Because of the need to improve the knowledge level of health professionals on IHS, the Acute Stroke Management Questionnaire (ASMaQ) was recently developed by an expert panel of neurologists in Malaysia (Sim et al. 2021). The instrument is the first of its kind to evaluate healthcare professionals' knowledge of acute stroke management (ASM) through three major components: general stroke knowledge (GSK), hyperacute stroke care (HSC) and advanced stroke management (Sim et al. 2021). A recent study by Albart et al. (2022) used the ASMaQ to evaluate healthcare professional knowledge of ASM and predictors of knowledge in a Malaysian population. The types of healthcare professionals included in this study were ‘medical practitioners’ and ‘allied healthcare professionals from various medical disciplines’ (Albart et al. 2022). Healthcare professionals who did not work with stroke patients daily had 2.67 times the odds of poor stroke knowledge compared to those working with stroke patients daily (*p* < 0.001). Additionally, non‐doctors had 3.46 times the odds of having poor stroke knowledge (*p* = 0.004). This finding is essential to consider, as most IHS strokes occur in cardiovascular and surgical areas, as explained above. Albart et al. (2022) did not differentiate nurses from other non‐doctors when reporting the results, so the level and predictors of stroke knowledge for nurses specifically are unknown.

## 3 Objective

Little is known on nurses' knowledge on stroke, specifically the nurse characteristics that may serve as predictors for their level of stroke knowledge. The purpose of this study is to identify nurse characteristics that may be predictors of stroke knowledge amongst acute care nurses.

## 4 Methods

### 4.1 Design

We conducted a non‐experimental, descriptive correlational study. We used multiple regression analyses to make inferences about the relationship between nurse characteristics (predictor variables) and stroke knowledge (response variable).

### 4.2 Sampling and Recruitment

The study took place at three community, Magnet‐designated hospitals that are part of the same healthcare system in an urban city in the Southeastern United States. Magnet designation is awarded to organisations who have proven nursing excellence and quality patient care (American Nurses Credentialing Center 2023). The two largest of the three hospitals are certified Joint Commission Primary Stroke Centers (PSC) (WakeMed 2022). The PSC certification is designated to hospitals that have taken extra efforts to ensure improved outcomes for stroke patients (The Joint Commission 2022). As of February 2022, the system employs about 3000 nurses. The largest of the three hospitals has 567 acute beds, the second largest has 208 acute beds, and the third has 61 acute beds (WakeMed 2022).

A convenience sample of RNs who provide direct patient care as their primary role at one (or more) of the hospitals was invited to participate. For this study, nurses who care for hospitalised patients will be referred to as acute care RN. Inclusion criteria included having an active RN licence, completion of orientation and working independently without a preceptor, and caring for hospitalised patients. Exclusion criteria included nurses working in the Neuro Intensive Care Unit (ICU), rehabilitation, paediatric units, labour and delivery and/or mother/baby units, and primarily in managerial roles. Neuro ICU nurses were excluded as it is believed their responses would provide skewed results as they are the hospitals' stroke nursing experts. Rehabilitation nurses were excluded as this hospital system designates rehabilitation as a non‐acute care setting, and there are many confounding variables for stroke symptom awareness and recognition in the rehabilitation setting. Nurses caring for paediatric, labour and delivery, and/or mother/baby populations were excluded as this study focuses on the knowledge level of stroke for adult patients. Nurses in managerial roles were excluded, as their primary role in the hospital is not to provide direct care for patients.

The targeted sample size was *N* = 106. This targeted sample size is based off the *N* > 50 + 8 m (*m* = number of predictor variables) recommendation by Pallant (2020) when conducting multiple regression analyses.

Exempt Certification was received in October 2022 from the East Carolina University Institutional Review Board (IRB) office prior to data collection, ensuring the protection of human participants and adherence to ethical standards. All RNs working within this hospital system were sent an email that included a secure link to an online survey for confidentiality, a flyer advertising the study, and pertinent information related to the study purpose, procedures, and inclusion and exclusion criteria. All email addresses used were via secured, hospital‐provided domains.

### 4.3 Instrument

The online survey consisted of three parts: demographic information, questions related to their stroke education and experience, and the ASMaQ.

#### 4.3.1 Demographic Survey

The demographic survey was developed by the principal investigator of the study. The first three questions of the demographic survey tested for inclusion criteria (licensed RN, provide care for adult, hospitalised patients, and do not work in the Neuro ICU, labour and delivery, and/or or mother/baby unit) to prevent ineligible participants from completing the full survey. The variable acute care RN, education, current practice setting, employment status and work experience were assessed via the demographic survey. Additionally, the demographic survey asked participants for birth year, gender and race/ethnicity.

#### 4.3.2 Nurse Stroke Education and Experience

This portion of the survey is a researcher‐developed instrument developed specifically for this study consisting of 10 multiple‐choice items and one drag item. Education and stroke knowledge, and professional and personal acute stroke experience were measured via this portion of the survey. Education was assessed through asking participants about their pre‐ and/or post‐licensure stroke training and the type of training, that is, lecture, clinical experience, simulation, informal on‐the‐job training, continued education module.

Professional acute stroke experience was assessed through asking participants about work on a stroke or a neuro‐specific unit, the length of time they worked there, in what capacity (RN, student, nurse aide) and the frequency, if ever, that the participant provides care for stroke patients in their current practice.

Personal acute stroke experience was assessed through asking participants if they have personally had or have a family member who has had a stroke, how the person is related to them and if and how this impacted their personal stroke knowledge.

Stroke knowledge will be partially measured using four items from this portion of the survey. Three multiple‐choice questions tested the participants' awareness of the code stroke process at the hospital in which they work, their comfort with the code stroke policies and procedure, and their confidence in activating the system. One question asked the participant to drag a line to represent their self‐perception of their stroke knowledge from none to expert (measured from 1 to 100). Stroke knowledge was also measured using the ASMaQ (Sim et al. 2021).

#### 4.3.3 ASMaQ

The response variable, stroke knowledge, was measured using the ASMaQ (Sim et al. 2021). Permission to use this tool for the purpose of this study was received from the ASMaQ author via email prior to data collection. The ASMaQ is a new, validated 29‐item questionnaire that tests and measures healthcare professionals' stroke knowledge (Sim et al. 2021). The ASMaQ content was developed through a review of stroke literature, pre‐existing stroke assessment scales, clinical practice guidelines of Malaysia that are based on AHA guidelines and expert opinion (Sim et al. 2021). Content validation of 110 questions was completed using an expert panel. After 79 questions were eliminated, 31 questions were used for psychometric testing on 158 participants to further evaluate the tool for validity (Sim et al. 2021). The authors also completed exploratory factor analysis, which narrowed the original 31 questions to 29. Cronbach's alpha value was 0.82, showing good reliability of the measurement tool (Sim et al. 2021). Cronbach's alpha for this study sample is 0.76.

The 29 items of the ASMaQ are broken up into three domains: GSK, HSC and ASM. All 29 items use a five‐point Likert scale ranging from one to five points (Sim et al. 2021). For 23 of the questions, five is the best response and one is the worst; however, six questions are negatively scored, meaning one is the best and five is the worst (Sim et al. 2021).

The GSK consists of 10 items with a possible score of 10–50, HSC consists of 9 items with a possible score of 9–45 and ASM consists of 10 items with a possible score of 10–50. The total possible ASMaQ score ranges from 29 to 145; the higher the score, the higher the stroke knowledge (Sim et al. 2021).

The STROBE checklist was used to strengthen the reporting of the results (von Elm et al. 2008).

### 4.4 Data Analyses

The first specific aim was to examine acute stroke knowledge amongst acute care nurses. Independent sample *t*‐tests and one‐way ANOVAs were used to compare ASMaQ mean total scores, domain scores and individual item scores between participant subgroups.

The second specific aim was to examine the predictability of categorical and quantitative study variables on acute stroke knowledge amongst study participants. A standard multiple regression, where all predictor variables were entered into the model at once, was used to explain how much of the variance in the dependent variable of total acute stroke knowledge was explained by the independent predictor variables (nurse characteristics). In addition, the regression indicated the relative contribution of each predictor variable.

Data analysis was completed using IBM (IBM Corp 2021) SPSS version 28.0.

## 5 Results

Of 219 eligible participants who began the survey, 196 participants completed in its entirety. Most participants were identified as female and White/Caucasian. Ages ranged from the early 20s to early 70s. As shown in Table 1, most participants' highest degree completed was a Bachelor of Science in Nursing (BSN). At the time of survey completion, 32.1% reported less than 5 years of experience as an RN and 11.2% of the participants had been an RN for greater than 25 years. Most participants worked full‐time RN hours (three, 12‐h shifts per week). A wide range of nursing specialties/practice areas were represented amongst the population including Emergency Department (ED), ICU, cardiac floor, medical/surgical floors and procedural areas. Table 1 provides full demographic information.

**TABLE 1:** Demographic characteristics of participants.

Characteristic	*n*	%
Gender		
Female	180	92
Male	15	8
Non‐binary	1	< 1
Age range		
< 23	2	1
23–32	69	35
33–42	55	28
43–52	36	18
53–62	30	15
63–72	4	2
Highest nursing degree		
ADN	39	20
BSN	131	67
MSN	23	12
DNP	2	1
PhD	1	< 1
Years of RN		
< 5	63	32
5–10	51	26
11–15	25	13
16–20	25	13
21–25	10	5
> 25	22	11
Nursing specialty/practice area		
Emergency Department	30	15
Cardiac ICU	35	19
Med/surg floor	45	23
Procedural area	23	12
Cardiac telemetry/stepdown	38	19
Surgical/trauma telemetry/stepdown	6	3
Neurological telemetry/stepdown	9	5
Float pool	10	5

*Note: N* = 196. All percentages are rounded to a whole number.

Table 2 presents stroke education and work experience amongst the nurse participants. Although most participants never worked on a stroke‐specific floor, almost all participants reported caring for a stroke patient in the past. The majority of participants received stroke education in their pre‐licensure training. Almost all received post‐licensure stroke education/training, most commonly through continuing education materials and informal, on‐the‐job training. Personal stroke experience (themselves, an immediate family member, extended family member or close friend) was reported by 42.9% of participants. Of those who had a personal stroke experience, almost a third reported that this experience affected their stroke knowledge. Of those with personal stroke experience who reported an effect on stroke knowledge, all reported an increase in knowledge from the experience.

**TABLE 2:** Nurse stroke education and experience.

Variable	*n*	%
Stroke floor experience		
Yes	50	26
No	146	74
Years of stroke experience^a^		
< 1	12	24
1–3	23	46
4–6	7	14
7–10	2	4
< 10	6	12
Cared for a stroke patient^b^		
Yes	188	96
No	8	4
Pre‐licensure stroke education		
Yes	128	65
No	25	13
Unsure	43	22
Type of pre‐licensure stroke education^c^		
One lecture	52	41
Multiple lectures	49	38
Simulation/Clinical experience	27	21
Post‐licensure education		
Yes	193	99
No	3	1
Type of post‐licensure stroke education^d^		
Continuing education	166	86
In‐service	107	55
Supervised practice/externship/residency	44	23
Informal on‐the‐job training	136	70
Competency training/new‐hire orientation	105	54
Independent reading/study	85	44
Personal stroke experience		
Yes	84	43
No	112	57
Effect of personal stroke experience on stroke knowledge^e^		
Increased knowledge	61	73
Decreased knowledge	0	0
No effect on knowledge	23	27
Familiar with code stroke policy/procedures		
Very aware	62	32
Aware	116	59
Neutral	12	6
Not familiar	6	3
Comfortable with activating code stroke		
Very comfortable	84	43
Comfortable	79	40
Neutral	21	11
Not comfortable	12	6
Confident in understanding code stroke for IHS		
Very confident	88	45
Confident	79	40
Neutral	21	12
Not confident	5	3
Never heard of it	1	< 1

*Note: N* = 196. All percentages were rounded.

^a^ Reflects the number and percentage of participants answering ‘yes’ to stroke floor experience.

^b^ Reflects the number and percentage of participants answering ‘yes’ to cared for stroke patient.

^c^ Reflects the number and percentage of participants answering ‘yes’ to pre‐licensure stroke education.

^d^ Reflects the number and percentage of participants answering ‘yes’ to post‐licensure stroke education.

^e^ Reflects the number and percentage of participants answering ‘yes’ to personal stroke experience. IHS = in‐hospital stroke.

Most participants were aware or very aware of the hospital‐specific policies and procedures related to code stroke processes, were very comfortable or comfortable with activating a code stroke and were very confident or confident in their understanding of the purpose of code stroke for IHS.

ASMaQ total scores ranged from 93 to 133 (*M* = 117.35, SD = 8.15). GSK scores ranged from 32 to 46 (*M* = 41.7, SD = 3.33), HSC scores ranged from 28 to 43 (*M* = 35.16, SD = 2.69), while ASM scores ranged from 29 to 50 (*M* = 40.48, SD = 4.59).

No statistically significant differences of stroke knowledge were found with any of the demographic variables. However, there were statistically significant differences on three survey items related to code stroke awareness and stroke knowledge scores. There were also differences found between ICU and ED nurses compared to all other practice settings.

### 5.1 Code Stroke and Stroke Knowledge

A one‐way between‐groups ANOVA was conducted to explore the impact of code stroke familiarity, comfort and understanding on stroke knowledge as measured by the ASMaQ. Participants were divided into three groups depending on their level of awareness, comfort or confidence of understanding of the code stroke measure.

There was a statistically significant difference at the *p* < 0.001 level in the mean ASMaQ total score for the three levels of nurse familiarity (very aware, aware, less than aware) with their hospital's code stroke policy and procedures: *F* (2, 193) = 22.35, *p* < 0.001. The effect size calculated using eta squared was large at 0.19. The Tukey HSD multiple comparison test indicated that the mean scores for all three groups were statistically significantly different from each other (very aware group [*M* = 122.1, SD = 6.04]; aware group [*M* = 115.82, SD = 7.97]; less than aware group [*M* = 110.83, SD = 7.56]).

There was also a statistically significant difference at the *p* < 0.001 level in the mean ASMaQ total score for the three levels of nurse comfort (very comfortable, comfortable, less than comfortable) with their ability to activate a code stroke if they assessed stroke symptoms in their patient(s): *F* (2, 193) = 27.93, *p* < 0.001. The effect size calculated using eta squared was large at 0.22. The Tukey HSD multiple comparison test indicated that the mean scores for all three groups were statistically significant from each other (very comfortable group [*M* = 121.5, SD = 6.75]; comfortable group [*M* = 115.41, SD = 7.61]; less than comfortable group [*M* = 111.42, SD = 7.37]).

Finally, there was a statistically significant difference at the *p* < 0.001 level in the mean ASMaQ total score for the three levels of confidence in understanding the purpose of code stroke for IHS (very confident, confident, less than confident): *F* (2, 193) = 22.25, *p* < 0.001. The effect size calculated using eta squared was large at 0.19. The Tukey HSD multiple comparison test indicated that the mean scores for the very confident group (*M* = 121.21, SD = 6.61) is significantly larger than that of the confident group (*M* = 114.62, SD = 7.67) and the less than confident group (*M* = 113.13, SD = 6.68); however, the difference in means for the confident and less than confident groups was not statistically significant (*p* = 0.619).

### 5.2 Practice Setting

Independent‐samples t‐tests were conducted to compare the stroke knowledge scores of nurses working in the ED and ICU compared to nurses working in all other practice settings. There was a statistically significant difference in general stroke knowledge scores (domain 1 of ASMaQ) for ED and ICU nurses (*M* = 42.49, SD = 2.81) compared to all other practice settings (*M* = 41.31, SD = 3.5; *t*(195) = 2.36, *p* = 0.019). The eta squared was small at 0.03. There was a statistically significant difference in advanced stroke management scores (domain 3 of ASMaQ) for ED and ICU nurses (*M* = 42.82, SD = 3.84) compared to all other practice settings (*M* = 39.33, SD = 4.51; *t*(195) = 5.35, *p* < 0.001). The eta squared showed a medium effect size at 0.13. There was also statistically significant difference in total ASMaQ scores for ED and ICU nurses (*M* = 120.97, SD = 7.18) compared to all other practice settings (*M* = 115.55, SD = 8.02; *t*(195) = 4.61, *p* < 0.001). The eta squared showed a medium effect size at 0.10.

There was a statistically significant difference in self‐identified stroke knowledge (scoring from 1 to 100) for ED and ICU nurses (*M* = 74.55, SD = 20.47) compared to all other practice settings (*M* = 60.92, SD = 20.83; *t*(195) = 4.34, *p* < 0.001). The eta squared showed a medium effect size at 0.09.

### 5.3 Post‐Licensure Stroke Training

Over 98% (*n* = 193) of the participants reported having some form of post‐licensure stroke education. The various forms of education methods include continuing education (84.7%), in‐service training (54.6%), supervised practice and/or externship (22.4%), informal on‐the‐job training and/or learning by doing (69.4%), competency training during new‐hire orientation (53.6%) and independent reading/study (43.4%). About a third (*n* = 63; 32.8%) of participants reported one or two of the listed post‐licensure education methods, while 67.2% (*n* = 129) reported three or more education methods.

An independent‐samples t‐test was used to compare the mean ASMaQ scores amongst participants who reported one or two post‐licensure education methods versus those who reported three or more. There were strong statistically significant differences between the two post‐licensure stroke education groups on GSK, ASM, HSK and total ASMaQ scores; see Table 3.

**TABLE 3:** Independent‐samples t‐test: post‐licensure stroke education and ASMaQ.

Variable	≤ 2^a^ *M*; SD	≥ 3^a^ *M*; SD	*t*	*p*
Total ASMaQ	113.35; 7.73	119.34; 7.68	5.07	< 0.001
Domain 1: GSK^b^	40.46; 3.76	42.28; 2.96	3.65	< 0.001
Domain 2: ASM^b^	33.94; 2.16	35.74; 2.75	4.57	< 0.001
Domain 3: HSC^b^	38.95; 4.51	41.32; 4.45	3.44	0.001
Self‐perception stroke knowledge	57.71; 21.56	69.29; 20.72	3.59	< 0.001

^a^ Number of post‐licensure stroke education methods.

^b^ GSK: general stroke knowledge; ASM: acute stroke management; HSC: hyperacute stroke care.

### 5.4 Predictability of Variables

Standard multiple regression was used to assess the predictability of various nurse characteristics on stroke knowledge as measured by the ASMaQ. Six of the nurse characteristic variables were entered into the multiple regression model. The six predictor variables, practice setting (ED/ICU vs. all others), code stroke awareness, code stroke activation comfort, code stroke confidence in understanding, self‐identified knowledge of ASM and self‐perception of stroke knowledge, were all found to be significantly correlated with the dependent variable (stroke knowledge as measured by the ASMaQ total score).

Preliminary analyses were conducted to ensure no violation of the assumptions of normality, linearity, multicollinearity and homoscedasticity. Variance inflation factor values were all small (< 10), indicating no problem with multicollinearity amongst the predictor variables. A p–p plot indicated no major deviations from normality for the residuals, and a scatterplot showed no residuals more than 3.3 or less than −3.3, indicating no outliers in the sample data.

The regression model (six predictor variables) explains 30.7% of the variance in the ASMaQ total scores, *F* (6, 189) = 13.93, *p* < 0.001. Practice setting (*p* = 0.013), self‐identified knowledge of ASM (*p* = 0.002) and self‐perception of stroke knowledge (*p* = 0.003) made statistically significant unique contributions to predicting ASMaQ total scores. Using the standardised beta (*β*) regression coefficients to assess the relative importance of the significant predictors, self‐identified knowledge of ASM (*β* = 0.25) and self‐perception of stroke knowledge (*β* = 0.24) were the most important predictors followed by practice setting (*β* = 0.16); see Table 4.

**TABLE 4:** Regression analysis summary of study variables predicting the ASMaQ total score.

Variable	*B*	SE *B*	*β*	*t*	*p*
Practice setting	2.76	1.11	0.16	2.50	0.013
Code stroke 1	1.82	1.91	0.06	0.95	0.343
Code stroke 2	1.47	1.63	0.07	0.90.	0.370
Code stroke 3	−1.12	1.63	−0.05	−0.69	0.490
Acute stroke management	5.54	1.74	0.25	3.18	0.002
Self‐perception stroke knowledge	0.09	0.30	0.24	3.01	0.003

*Note: R* ^2^ = 0.307 (*N* = 196, *p* < 0.001). *B* = unstandardised multiple regression coefficient. SE = standard error. *β* = standardised multiple regression coefficient. Code stroke 1 = I am familiar with policy/procedures of this hospital stroke code. Code stroke 2 = I would feel comfortable activating a stroke code. Code stroke 3 = I am confident I understand the purpose of stroke code for IHS. Acute stroke knowledge: 1 = very poor to 5 = very good. Self‐rating of stroke knowledge: 100‐point scale from 1 to 100.

## 6 Discussion

Although the results did not indicate specific demographic variables that may predict stroke knowledge, other variables such as practice setting, methods of post‐licensure stroke education, code stroke familiarity, confidence and understanding were all predictive of the overall stroke knowledge. Nurses working in the ED and ICU were found to have higher levels of stroke knowledge when compared to nurses working in all other practice settings. This could be attributed to higher frequency and intensity of educational initiatives amongst ED and ICU nurses, as patients often experience higher acuity and code stroke educational initiatives have primarily focused amongst ICU nurses (George et al. 2017). Although important that ED and ICU nurses have a high level of stroke knowledge, half of IHS occur on cardiology/cardiovascular surgery practice areas (Yoo et al. 2016), deeming a high importance of educational initiatives for nurses caring for these patient populations.

Nurses who reported experiences with three or more modalities of post‐licensure stroke education were found to have higher levels of stroke knowledge than those who only received one or two education methods. These reported methods include continuing education, in‐service training, supervised practice and/or externship, informal on‐the‐job training and/or learning by doing, competency training during new‐hire orientation and/or independent reading/study. This shows that various methods of training and reinforcement of learning using various teaching styles and methods are effective in increasing nurses' knowledge amongst this population. This could be attributed to various learning styles and the variance in effectiveness depending on the individual nurse. This survey only allowed the participants to choose from a selection of pre‐labelled education methods and may not be inclusive of all the unique stroke education methods that the nurses have received that contribute to their overall stroke knowledge.

Nurses who reported familiarity with the hospital's code stroke policies and procedures, comfort in activating a code stroke if needed and understanding of the purpose of code stroke for IHS also were found to have higher levels of stroke knowledge. It is important for nurse leaders and educators to understand the value in including hospital‐specific policies and procedures when designing stroke education.

Nurses who are self‐identified as having a high level of stroke knowledge were also validated by their ASMaQ stroke knowledge scores. This shows that nurses amongst this population have a high level of self‐awareness and were correct in their personal assumptions of their stroke knowledge.

‘The Great Resignation’ has been coined to describe today's workforce and nursing climate of high turnover rates and increased number of travel nurses and transient staff (Laskowski‐Jones and Castner 2022). Retaining experienced and senior nurses is essential to high‐quality patient care, including stroke care (Laskowski‐Jones and Castner 2022). However, due to record‐breaking turnover rates and the result of nurse burnout, predictions show that new‐graduate nurses will soon become an even larger proportion of the nursing workforce (Laskowski‐Jones and Castner 2022). This creates instability and gaps in support and education for the new graduates as well as a lack of balanced experience levels amongst the nursing units and areas. Soon, if not already, there will be a vacancy of preceptors, mentors and experienced nurses to provide hands‐on support to new‐graduate nurses or nurses transitioning to new practice areas. Having a more detailed understanding of the factors and lived experiences that have shaped nurses' stroke knowledge and ability to carry out code stroke processes will help educators' creativity in providing effective stroke education.

With the record‐breaking nurse turnover rates, it is important for nurse managers and educators to have a good understanding of new nurse (both new graduates and experienced nurses) baseline stroke knowledge level upon hire. The ASMaQ is a quick, short survey that takes less than 5 min to complete. Of the survey participants who began the ASMaQ portion of the survey, 94% finished the 29‐item questionnaire. This shows very low attrition and respondent fatigue. Nurse managers and educators could use the ASMaQ to assess baseline stroke knowledge at hire to create a tailored stroke orientation experience. This has the potential to be a cost‐saving initiative as nurses would only be receiving the stroke education that is filling the gaps of what is not already known. Cost saving initiatives are imperative in today's healthcare climate, as an increase in patient acuity and volumes has led to an overall increase in healthcare costs (American Hospital Association 2022).

The American Hospital Association (2022) has reported an increase in patient acuity and an increase in the average hospital length‐of‐stay by 10% since pre‐pandemic. This has led to an increased hospital demands and patient volumes, which in turn has led to heterogeneous patient care units. Patients are no longer strictly assigned to specific practice settings due to their diagnoses but rather assigned to the quickest availability to lessen lengthy holding periods in the ED. However, this leads to nurses caring for a wide variety of patients and conditions that they are not as familiar with or continually educated on. This is suggested in our survey results, as only about a quarter of participants reported experience working on a stroke‐specific floor, but almost all reported caring for a stroke patient in the past. This reinforces the importance of standardised stroke education for all nurses caring for hospitalised patients, not just nurses working in the ICU or ED.

### 6.1 Recommendations

Future studies should consider exploring the lived experiences of nurses through an interview or focus group format to have a better understanding of the nonquantifiable factors that nurses feel have strengthened their stroke knowledge. Certain educational initiatives are not easily captured via survey such as mentorship, an environment of teamwork, grand rounds, etc. These educational experiences could shape a nurse's understanding of stroke and stroke care and contribute to the overall stroke knowledge and stroke skillset. Knowing this information could help nurse educators and managers appropriately prepare nurses to care for not only stroke patients but also all hospitalised patients that may be at risk for an IHS. This is imperative as nurses continue to navigate care of an increasingly complex patient population while also battling the effects of the Great Resignation.

### 6.2 Limitations

Due to the convenience sampling method, results may not be generalisable to the general population. Also, since this study was observational in nature, there is an inability to support causal relationships (Polit and Beck 2021). Data collection and the testing of nurses' stroke knowledge occurred online and without supervision. Because of this, the study team was unable to verify that all participants answered the items independently and without assistance.

This study surveyed nurses from one hospital system, serving an urban Southeast population. Future studies should consider using the ASMaQ or testing the stroke knowledge amongst a wider range of nurses, including those in rural areas.

## 7 Conclusion

ICU and ED nurses had higher stroke knowledge scores when compared to nurses in other practice settings. Nurses who received three or more modalities of post‐licensure stroke education had higher levels of stroke knowledge compared to those who only had one or two. Nurses who reported familiarity, comfort and confidence in understanding their hospital's code stroke process had higher levels of stroke knowledge. The findings of this study reinforce the importance of continued stroke education for nurses in all departments. Increasing the stroke knowledge of nurses working in all practice settings within a hospital system will improve the timeliness and appropriateness of response when IHS occur.

## Author Contributions

Catherine T. Leach: conceptualisation, data curation, formal analysis, investigation, methodology, project administration, visualisation, writing – original draft. Linda P. Bolin: conceptualisation, methodology, project administration, resources, supervision, writing – review and editing. Melvin S. Swanson: data curation, formal analysis, methodology, writing – review and editing. Ashley E. Burch: data curation, methodology, validation, writing – review and editing. Patricia C. Woltz: resources, writing – review and editing. There is a statistician on the author team: Melvin Swanson, PhD.

## Conflicts of Interest

The authors declare no conflicts of interest.

## Data Availability

The data that support the findings of this study are available from the corresponding author upon reasonable request.
